# Host Specificity and Differential Pathogenicity of *Pectobacterium* Strains from Dicot and Monocot Hosts

**DOI:** 10.3390/microorganisms8101479

**Published:** 2020-09-26

**Authors:** Nirmal Khadka, Janak Raj Joshi, Noam Reznik, Nofar Chriker, Adi Nudel, Einat Zelinger, Zohar Kerem, Iris Yedidia

**Affiliations:** 1 Department of Biochemistry, Food Science and Nutrition, The Robert H. Smith Faculty of Agriculture, Food and Environment, The Hebrew University of Jerusalem, POB 12, 7610001 Rehovot, Israel; nirmal.khadka@mail.huji.ac.il (N.K.); janakra.joshi@mail.huji.ac.il (J.R.J.); nofar.tsriker@mail.huji.ac.il (N.C.); nudel.adi@gmail.com (A.N.); einat.zelinger@mail.huji.ac.il (E.Z.); 2 Institute of Plant Sciences, Agricultural Research Organization, Volcani Center, POB 15159, 752880 Rishon Lezion, Israel; noamr@volcani.agri.gov.il

**Keywords:** host extracts, host specificity, *Pectobacterium*, plant defense, differential virulence

## Abstract

Recent phylogenetic studies have transferred certain isolates from monocot plants previously included in the heterogeneous group of *Pectobacterium* *carotovorum* (Pc) to a species level termed *Pectobacterium aroidearum*. The specificity of *Pectobacterium* associated infections had received less attention, and may be of high scientific and economic importance. Here, we have characterized differential responses of *Pectobacterium* isolates from potato (WPP14) and calla lily (PC16) on two typical hosts: *Brassica oleracea* var. *capitata* (cabbage) a dicot host; and *Zantedeschia aethiopica* (calla lily) a monocot host. The results revealed clear host specific responses following infection with the two bacterial strains. This was demonstrated by differential production of volatile organic compounds (VOCs) and the expression of plant defense-related genes (*pal*, *PR-1*, *lox2*, *ast*). A related pattern was observed in bacterial responses to each of the host’s extract, with differential expression of virulence-related determinants and genes associated with quorum-sensing and plant cell wall-degrading enzymes. The differences were associated with each strain’s competence on its respective host.

## 1. Introduction

Pectobacteria are plant pathogens that cause soft rot, black leg and wilt in numerous plant species in more than 35% of angiosperm plant orders. Host specificity has never been considered to be an attribute of pectobacteria, although some host specificity has been described for *Pectobacterium atrosepticum* and *P. wasabiae* in connection with adaptation to a cooler climate and relatively narrow (but not exclusive) host range, that is potato (*Solanum tuberosum*) and Japanese horseradish (*Eutrema japonicum*), respectively [1,2,3]. In terms of host range, *P. carotovorum* probably has the widest host range and geographical distribution, with strains of this species isolated from various substrates including plant surfaces and tissues, soil, water and the gut and surfaces of invertebrates [4]. Another broad host range species is *P. brasiliense*, which is associated with potato grown in relatively warm regions in Brazil, Israel, South Africa and United States, but may also infect other crops [5]. With the ever-increasing availability of genomic sequences, several taxonomic changes have been made, including the suggestion to transfer *Pectobacterium* and *Dickeya* into the family Pectobacteriaceae [6]. According to the present taxonomy, the genus now includes 18 species: *P. actinidiae, P. aquaticum, P. aroidearum, P. atrosepticum*, *P. betavasculorum, P. brasiliense, P. cacticida*, *P. carotovorum*, *P*. *odoriferum*, *P. parmentieri, P. parvum, P. peruviense, P. polaris, P. polonicum, P. punjabense, P. versatile, P. wasabiae* and *P. zantedeschiae,* [4,7,8,9,10,11]. In spite of the clarifying taxonomy, the mechanisms underlying this natural segregation are largely unexplored. The last decade, has proved that *P. carotovorum* had been serving as a catchall designation for pectobacterial isolates that did not fall within any other specific taxon [12]. The use of multi-locus sequence analyses (MLSA) revealed the heterogeneity of *P. carotovorum*, and the insufficient resolution of former taxonomic tools. One particular example was the isolates of *P. carotovorum* obtained from monocot hosts. These were reported to be more virulent on monocot plants than on dicot plants, presented typical amplified fragment length polymorphism (AFLP) profiles and distinct banding pattern in intergenic transcribed spacer (ITS) PCR analysis [13]. The combination of molecular patterns with MLSA and recombinase polymerase amplification analyses indicated that these monocot isolates represent multiple species in the genus, *P. aroidearum, P. zantedeschiae, P. betavasculorum,* and *P. wasabiae* [12,14,15]. Atypical infections have also been reported in other monocots including *Allium cepa* [16], *Zantedeschia* spp. [17,18], *Dieffenbachia* spp., *Scindapsus aureus* [19], *Ornithogalum dubium* [1] and *Pinellia ternate* [20]. Past studies have shown that, under laboratory conditions, a single strain may infect several plant species, including crop plants such as potato, carrot (*Daucus carota* subsp. *sativus*), cabbage, tomato (*Solanum lycopersicum*) and peppers (*Capsicum annuum*) [1,4]. Host adaptation (specialization) has been suggested as one of the forces underlying the course of *Pectobacterium* evolution, but this hypothesis has not been extensively examined [13,16,21]. Greater fitness for infection of a host or a limited group of hosts was suggested mainly based on phylogenetic comparisons, geographical distribution and associations with the taxonomy of the hosts [22,23]. Only a few studies have focused on physiology or virulence patterns of different strains on taxonomically diverse hosts [13,17].

Here, host specialization was tested using two *Pectobacterium* strains, a ‘monocot strain’ (PC16, isolated from *Zantedeschia*) and a ‘dicot strain’ (WPP14, isolated from potato) on calla lily (*Zantedeschia aethiopica*) and cabbage (*Brassica oleracea* var. *capitata*). Host attributes such as volatile emissions and expression of defense-related typical genes were evaluated and used to explore the compatibility of the two bacterial strains with the two hosts. Similarly, bacterial responses to each of the hosts or host extracts were assessed in terms of their pathogenicity and virulence. The results support a compatible interaction between certain hosts and their respective monocot/dicot *Pectobacterium* isolates.

## 2. Materials and Methods

### 2.1. Bacterial Strains, Plants and Growth Media

*P. aroidearum* strains PC1 and PC16, which were isolated from *Ornithogalum dubium* and *Z. aethiopica*, respectively, and *P. carotovorum* strain WPP14 and *P. brasiliense* strain Pb1692, which were originally isolated from potato, were used for the infection assays. PC1 and PC16 were identified as typical monocot strains, whereas Pb1692 and WPP14 have been defined as common dicot strains [13]. Biochemical and molecular assays were performed with WPP14 and PC16 as representatives of the two groups, mainly as *P. carotovorum* represents the widest host range in the genus. Two classic hosts of *Pectobacterium* were used in the study: cabbage as a dicot host and calla lily as a monocot host. The bacterial strains were cultivated at 28 °C in lysogeny broth (LB; Difco Laboratories, Detroit, MI, USA) or in minimal medium (MM) prepared as described previously [24].

### 2.2. Virulence Assays

Virulence assays were performed in calla lily leaves or cabbage leaves using the *Pectobacterium* strains described above (i.e., PC1, PC16, Pb1692 and WPP14). Potato tubers were not used for the infection analyses, as they are too easily infected by all strains. To assess infection symptoms, calla lily and cabbage leaves were infiltrated with 100 µL bacterial cultures, that had been grown overnight, centrifuged at 14,000 rpm/3 min and re-suspended in sterile double-distilled water (DDW) at cell density of 10^7^ colony forming units (cfu)/mL. As a negative control, leaves were infiltrated with the same volume (100 µL) of sterile DDW and kept at 28 °C for 24 h, with leaf petioles dipped in water. Finally, the appearance of water-soaked lesions was recorded. Leaf-disc infection assays were carried out in calla lily and cabbage leaves with each of the strains, PC16, PC1 or WPP14 and Pb1692 as described previously [13,25]. Other experiments to unravel host specificity were performed using PC16 and WPP14 strains.

### 2.3. Scanning Electron. Microscopy (SEM)

SEM micrographs were taken on calla lily and cabbage leaves following disinfection in 0.7% sodium hypochlorite for 15 min and a double wash with sterile DDW. Bacterial cultures (PC16 and WPP14) that had been grown overnight were centrifuged and re-suspended in sterile DDW at 10^8^ cfu/mL and 10 µL of that suspension were placed on the leaf surface and incubated at 28 °C for 3 h inside an airtight box. Leaf samples (4 × 4 mm), from bacterial inoculation site, were gently rinsed with DDW, then fixed in 70% ethanol overnight, followed by dehydration with 90%, 95%, 100% ethanol for 1 h each and a second dehydration with 100% ethanol. Then, the samples were dried on a K850 critical-point dryer and coated with gold-palladium alloy in a Q150T ES turbo-pumped sputter coater, following the manufacturer’s instructions (Quorum Technologies Ltd., East Sussex, UK). Finally, samples were observed under SEM, JSM-7800F (JEOL Inc., Peabody, MA, USA). Experiment was performed with five biological repeats and 10 replicates for each strain-host combination. The micrographs are representative photos of the experiment. In addition, 5 leaves were inoculated as above, and 3 h post inoculation were washed gently in DDW, and sections (4 × 4 mm) were excised and grind in 200 µL sterile DDW, serial dilutions were made and plated onto LB agar for CFU assessment.

### 2.4. Analysis of Volatile Organic Compounds (VOCs)

Leaf samples were treated by infiltrating bacterial cultures as detailed above and incubated in airtight jars at 28 °C. Volatile compounds produced during 24 h were adsorbed onto a solid-phase microextraction (SPME) fiber divinylbenzene/carboxen/polydimethylsiloxane (DVB/CAR/PDMS) that was inserted through septum into each jar for the final 1 h. Every SPME then served to inject the adsorbed VOCs into the injection port kept on 250 °C, of a HP Agilent series 4890D gas chromatograph, equipped with a flame ionization detector (GC-FID), DB-5MS column (30 m × 0.32 mm, 0.5 µm film thickness; J & W Scientific, Folsom, CA, USA) operated in split mode. For calla lily samples, initial temperature of the oven was held at 50 °C for 1 min, raised to 220 °C at a rate of 8 °C/min. For cabbage samples, the initial temperature of the oven was held at 50 °C for 1.5 min, and then increased to 200 °C at a rate of 5°C/min, and to 280 °C at a rate of 10°C/min. Helium served as carrier gas, injection port temperature was set to 250 °C and detector temperature to 300 °C. Gas chromatography-mass spectrometry (GC-MS) was carried out on Agilent GC 7890A and 5975C MSD, operated as above. Chromatographic separation was carried out on a Rxi-5MS capillary column (30 m × 0.25 mm, 0.25 µm film thickness; Restek, Bellefonte, PA, USA). Mass spectra were acquired in positive electron-impact (EI) scan mode (m/z 20–350). Identification of the VOCs was performed by comparing their mass spectra and retention time with the NIST/EPA/NIH Mass Spectral Database (NIST 05, National Institute of Standards and Technology, Gaithersburg, MD, USA). Further, six commercially available compounds (methyl acetate, ethyl acetate, ethanol, isopentyl alcohol, isobutyl acetate, 4-methyl heptane) were purchased (Sigma-Aldrich, Rehovot, Israel) and chromatographed using GC-FID to support the identification, as described above.

### 2.5. Plant. Extracts

To extract phenolic compounds, cabbage and calla lily leaf samples were crushed in liquid nitrogen using mortar and pestle, and 200 mg were transferred into a 2 mL tubes, to which acidified aqueous methanol (1 mL, methanol:DDW:acetic acid, 11:5:1 *vol*/*vol*, respectively) was added. The supernatant was collected following vortex (1 min) and sonication (30 s 2×), and centrifugation (14,000 rpm/10 min/4 °C). The pellet was re-extracted in acidified aqueous methanol as above, and both extracts were pooled together and fractionated (2×) against hexane (0.6 mL hexane/1 mL extract), to remove hydrophobic residues (chloroplasts and cell membranes). The upper phase were discarded and the extracts were used immediately or stored at –80 °C for future use.

### 2.6. Growth Assessment

Bacterial growth in the presence of the plant extract was tested with *Pectobacterium* strains PC16 and WPP14. Both strains were grown overnight at 28 °C in 4 mL LB under continuous shaking at 150 rpm in a TU-400 incubator shaker (M.R.C. Ltd., Holon, Israel). The suspensions were then diluted in fresh LB to a concentration of 5 × 10^6^ cfu/mL and 200 μL of it were added to 96-well microtiter plates containing increasing volumes of the host extracts or extraction solution (control), that were dried in a laminar flow hood prior to adding the suspensions. Plates were incubated at 28 °C with continuous shaking and growth was assessed every hour for 16 h by measuring optical density at 600 nm using a micro-plate reader (Spectra MR, Dynex Technologies, Chantilly, VA, USA).

### 2.7. Exoenzyme Activity

Exoenzymes are the main factor responsible for the development of disease symptoms. Of these, pectate lyase (Pel) and polygalacturonase (Peh) are the most studied, but proteolytic enzymes (Prt) and cellulases (Cel) also play a role. Here, the levels of Pel, Peh and Prt were evaluated in the presence of a non-lethal volume (less than 50% growth inhibition) (40 µL extract/200 µL LB) of host extract in semi-quantitative enzymatic activity assays carried out in plates containing different substrates (Pel assay medium: 1% polygalacturonic acid, 1% yeast extract, 0.38 µM CaCl_2_, 0.8% agarose and 100 mM Tris-HCl pH 8.5; Peh assay medium: 1% polygalacturonic acid, 1% yeast extract, 2.2 mM EDTA, 0.8% agarose and 110 mM sodium acetate, pH 5.5; Prt assay medium: 3% gelatin, 0.4% nutrient broth and 0.8% agarose), as described previously [26]. *Pectobacterium* strains were exposed to 1:4 dilution ratio of dried host extracts resuspended in 4 mL LB, for 8 h at 28 °C with continuous shaking. Eight hours reflect the exponential growth phase of the bacteria. Then, 4 mm holes were poked in the medium plates containing substrates and those holes were filled with filter sterilized supernatants (20 µL) of the grown cultures and incubated at 28 °C for 18 h. Pel and Peh plates were then submersed in 4N HCl to observe the haloes created by the enzymes, while Prt haloes were observed directly. The haloes were measured and compared with control (extraction solution).

### 2.8. Biofilm Formation

Biofilm formation was evaluated in glass test tubes, using crystal violet staining, as described previously [27]. Briefly, 100 µL of host extracts (i.e., calla lily and cabbage extracts) and extraction solution (control) was added to sterile and clean glass test tube and dried under laminar. Overnight grown *Pectobacterium* strains were adjusted to a concentration of 2 × 10^6^ cfu/mL, using fresh LB. Five hundred microliters of the bacterial culture were transferred to the test tubes containing dried extracts and extraction solution. The tubes were then incubated at 28 °C for 72 h. Next, the bacterial suspensions were carefully discarded, and test tubes were washed with deionized water. Following washing, 500 µL of 0.1% crystal violet was added to each test tube and incubated (25 °C, 15 min) to stain the biofilm. Staining was followed by washing the tubes with deionized water twice. Finally, 500 µL of 30% acetic acid, was added to the tubes and incubated at 25 °C for 15 min, following absorbance measurement at 550 nm in a micro-plate reader (Spectra MR).

### 2.9. Total RNA Extraction and cDNA Preparation

#### 2.9.1. Extraction of RNA from the Pectobacteria

*Pectobacterium* strains were grown overnight at 28 °C in LB medium under continuous shaking. The bacteria were centrifuged at 14,000 rpm for 3 min, washed and then transferred to fresh LB to a final concentration of 10^6^ cfu/mL, with a non-lethal volume (40 µL extract/200 µL LB) of the phenolic extract of cabbage or calla lily or extraction solution (control). The suspensions were then incubated at 28 °C under continuous shaking (150 rpm) for 8 h. RNA was extracted from 2 mL of the culture using the EZ-RNA II kit (Biological Industries, Kibbutz Beit Haemek, Israel), following the manufacturer’s instructions.

#### 2.9.2. Extraction of RNA from Calla Lily and Cabbage

Calla lily and cabbage leaves were rinsed twice in sterile DDW, wiped gently with absolute ethanol and infiltrated with bacterial cultures that had been grown overnight (100 µL), followed by centrifugation and resuspension in sterile DDW to a final cell density of 10^7^ cfu/mL and then incubated at 28 °C for 24 h. The control treatment included leaves that were infiltrated with the same amount of sterile DDW. Total RNA was extracted from leaf piece (100 mg, tissue around necrotic area) using RNA buffer (10 mM Tris-HCl pH 8.0; 1 mM LiCl; 0.2 mM EDTA and 1% LiDS) and hot phenol. Briefly, leaf pieces were ground to a fine powder in liquid nitrogen and mixed with a pre-heated mixture of RNA buffer and phenol (80 °C until the tissue became transparent, 0.6 mL per leaf piece). The extract was then mixed and incubated at 80 °C for 5 min. Then, 650 µL of chloroform: isoamyl alcohol (24:1) was added and the mixture was mixed well for 3 min. The samples were centrifuged at 13,000 rpm for 5 min. Three phases were obtained, and the upper phase was transferred to a clean tube and placed on ice. This step was repeated twice with 1 mL chloroform and isoamyl alcohol mixture to attain a clean upper phase, which was transferred to the clean tube. Next, cold 10 M LiCl was added to the tube in a ratio of 3:1 (sample:LiCl). The solution was mixed gently and incubated at −20 °C overnight. The next day, samples were thawed on ice and centrifuged at 4 °C and 12,000 rpm for 30 min to obtain crude RNA. The RNA pellet was washed twice with 1 mL of 70% ethanol and then dried for 30 min. The pellets were then dissolved in 90 µL DEPC-treated DDW. Samples were treated with RNase-free DNase (Thermo Scientific, Waltham, MA USA) to remove any residual DNA. Further cleaning was done using a Zymo-Spin IIC column with RNA binding buffer, RNA preparation buffer and RNA wash buffer (Zymo Research, Irvine, CA, USA).

#### 2.9.3. cDNA Preparation

For cDNA synthesis, a minimum of 1 µg of total RNA was reverse transcribed in a reaction mixture of 20 µL using a cDNA synthesis kit (Applied Biosystems, Foster City, CA, USA) following manufacturer’s instructions. The cDNA reverse-transcription reaction was performed using a programmable thermal controller (MJ Research, Quebec, Canada) programmed to one cycle at 37 °C for 60 min, followed by inactivation at 95 °C for 5 min. The obtained cDNA was stored at −20 °C for future use.

### 2.10. Primer Design and mRNA Quantification by Real-time qRT-PCR

Several virulence genes of *Pectobacterium* have been identified and well characterized. The most recognized are genes related to plant cell wall-degrading enzymes (PCWDEs), quorum-sensing and the type III secretion system. Accordingly, we examined the expression levels of *pel* and *peh* (representative PCWDE genes), *expI* and *expR* (QS system genes), *hrpL* (representative of T3SS) and *araF* (a representative membrane-transporter gene). The primer specificity for the aforementioned gene has already been validated previously by Joshi et al. [28], the same primers were used in this study. Similarly, the expression levels of defense-related genes (lipoxygenase *lox2*, pathogenesis-related gene *PR-1*, phenylalanine ammonia lyase *pal* and aspartate aminotransferase *ast*) in calla lily and cabbage were evaluated. Primers for these genes in calla lily were designed using Primer 3.0 (http://bioinfo.ut.ee/primer3-0.4.0/) software as explained [28] and detailed in Table 1. Gene sequences of calla lily is not available in the database, therefore cDNA sequences of defense related genes were sequenced by our lab and deposited at figshare.com (DOI 10.6084/m9.figshare.12326495). Sequences of the same genes for cabbage were obtained from the Joint Genome Institute (JGI) database (https://phytozome.jgi.doe.gov/pz/portal.html) and primers were designed as explained above and detailed in Table 1. The specificity and quality of the primers were tested using serial dilution of cDNA mix from all treatments and data were translated into standard curve. Specifically, all primers were tested for their specificity (single product amplification, observed as single melting curve), efficiency (90–105% observed) and correlation coefficient R^2^ (>0.98 observed). Primers, that passed quality check, were used for qRT-PCR reactions using the Step One Plus Real-Time PCR system (Applied Biosystems Inc. Foster City, CA, USA), with reaction details mentioned as in Joshi et al. [28]. Finally, data were analyzed by the comparative C_t_ (ΔΔC_t_) method, relative to control (bacteria cultured in sterile water), with expression normalized to the expression of the reference gene *recA* for bacteria, actin for calla lily, and tubulin for cabbage. The reference genes were selected based on their expression consistency under all conditions.

### 2.11. Statistical Analysis

**Figure 1:**
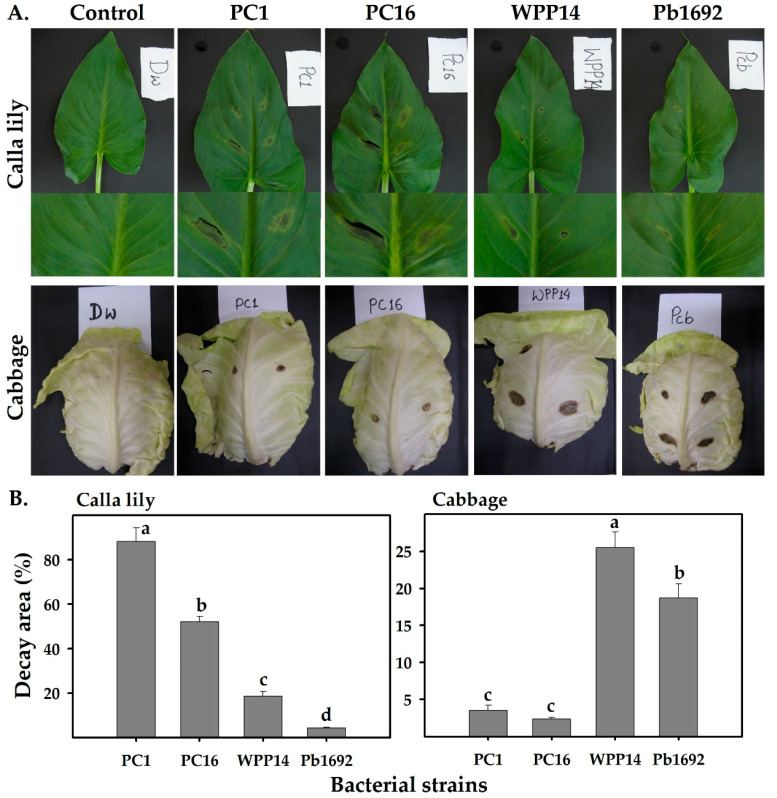
Disease symptoms on calla lily or cabbage leaves in response to inoculation with *Pectobacterium* strains isolated from monocot or dicot hosts. (**A**) *Pectobacterium* strains PC1 and PC16 (*P. aroidearum* isolated from *Ornithogalum dubium* and *Zantedeschia aethiopica*, respectively), Pb1692 and WPP14 (*P. brasiliense* and *P. carotovorum*, both isolated from potato) were infiltrated into calla lily or cabbage leaves (100 µL, 10^7^ cfu/mL sterile DDW) and disease symptoms were evaluated 24 h after inoculation. (**B**) Leaf discs assessment of calla lily or cabbage inoculated with the same strains as above (10 µL of 10^8^ cfu/mL). Infection was measured as percentage of decayed tissue at 15 h after inoculation. The full-leaf experiment (A) was repeated twice with five replicates and the leaf-disc experiment (B) was repeated twice with 20 replicates for each strain/host combination. Bars represents mean ± standard errors (SE) of decayed area (%). Bars not labeled with the same letter (a–d) are significantly different from each other (one-way ANOVA with post hoc Tukey HSD test, *p* < 0.05).

Leaf discs infection area and enzymatic assays were measured using ImageJ software, version 1.52e (National Institutes of Health, USA). Statistical comparisons were made using one-way analysis of variance (ANOVA) by JMP-Pro software, version 13.0 (SAS Institute Inc., Cary, NC, USA). When ANOVA yielded significance (*p* ≤ 0.05), Tukey–Kramer HSD test or Student’s *t*-test were performed. Tukey–Kramer HSD test was used for Figures 1B, 4, 6, 7, 8 and Figure S4; Student’s *t*-test was used for Figure S2. Data presented were means ± standard errors (SE). Graphs were generated using Sigma Plot Version 10.0 software (Systat Software, San Jose, CA, USA).

## 3. Results

### 3.1. Virulence of Pectobacterium Strains on Different Hosts

We examined the abilities of four *Pectobacterium* strains (PC1, PC16, Pb1692 and WPP14) to infect cabbage and calla lily leaves. Both PC1 and PC16 produced visible necrosis symptoms on calla lily leaves. However, on cabbage leaves, those isolates produced limited symptoms. In contrast, Pb1692 and WPP14 infected cabbage leaves but produced hardly any symptoms on calla lily leaves (Figure 1A). Similar infection patterns were observed in a leaf-disc assay, in which the leaf disc was pierced at its center and the bacterial suspension was applied to that spot. This assay allowed the evaluation of tissue decay caused by PC16, PC1, Pb1692 and WPP14 on both hosts and revealed significantly greater decay following each strains infection of its “favored” host. That is, PC16 and PC1 caused more decay on calla lily and Pb1692 and WPP14 caused more decay on cabbage (Figure 1B) (Figure S1).

### 3.2. Attachment of Pectobacterium Strains to Host Leaf Surfaces

**Figure 2:**
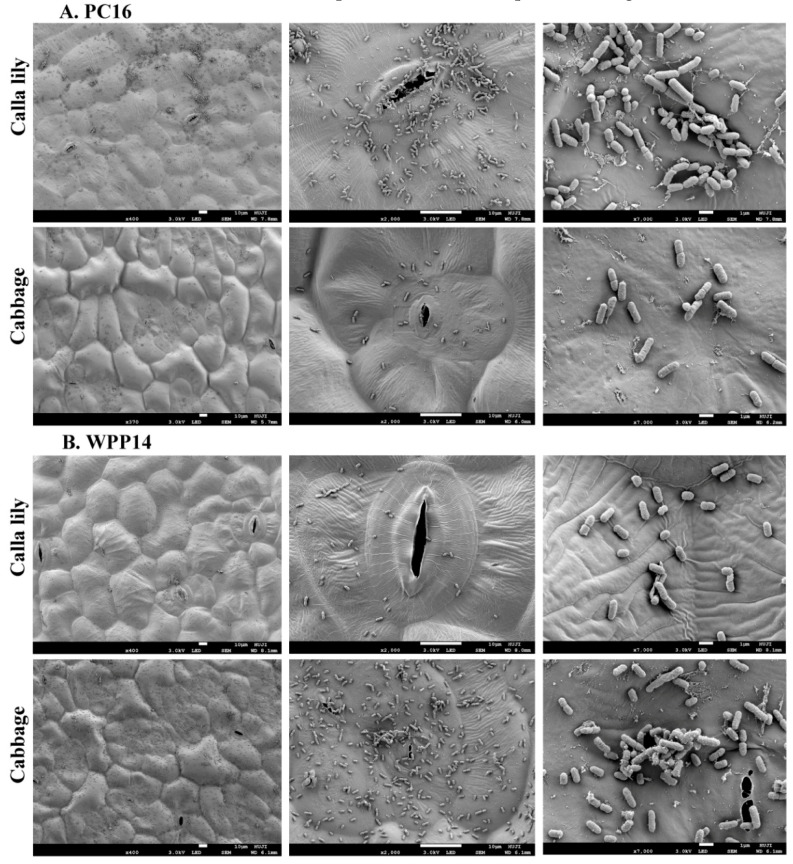
SEM micrographs of (**A**) *P. aroidearum* PC16 and (**B**) *P. carotovorum* WPP14 on the leaf surfaces of calla lily and of cabbage. The micrographs were obtained 3 h after inoculation (10 µL, 10^8^ cfu/mL) onto the host leaf surfaces. The colonization patterns were examined on 10 leaves for each plant. SEM images are presented (left to right) magnification 400×, bar =50 µm; 2000×, bar = 10 µm; magnification 7000×, bar = 1 µm.

In order to study early colonization patterns of the strains, we have focused on PC16 and WPP14 on the two hosts (calla lily and cabbage). Bacterial suspensions (10 µL, 10^8^ cfu/mL DDW) were applied by pipetting onto the leaf surfaces in an airtight box. SEM micrographs of leaf-surface colonization were obtained 3 h post inoculation, revealing distinctive patterns of attachment and proliferation for each strain on the two hosts. PC16 exhibited greater proliferation and attachment on the surface of calla lily leaves, whereas WPP14 performed better on cabbage (Figure 2). The performance of each strain on its non-favored host was poor, with less biofilm initiation, clustering and/or colonizing of cells. In addition to SEM, the inoculated tissues were grinded and serially diluted for bacterial enumeration. The results revealed significantly higher cell numbers for each strain on its competent host. Bacterial cell numbers (CFU/mL) 3 h post inoculation are provided (Figure S2).

### 3.3. Production of VOCs by Calla Lily and by Cabbage in Response to Inoculation of Pectobacterum

VOCs are produced by plants as part of the early reaction to and interaction with microorganisms [29,30]. VOCs emitted from calla lily or cabbage leaves following inoculation with either PC16, WPP14 or DDW (mock-inoculated control) were contained for 24 h. Thermal desorption served to release adsorbed VOCs that were produced for 24 h by the treated leaves in a sealed glass jar for 24 h and adsorbed onto SPME fibers.

**Figure 3:**
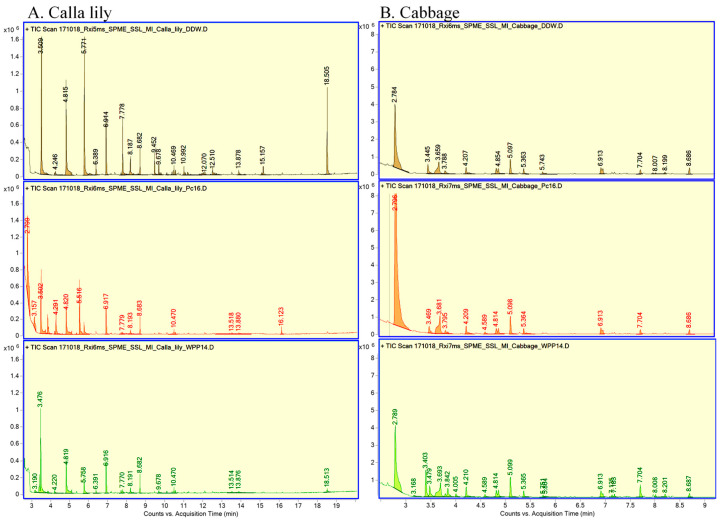
Chromatograms of volatile organic compounds (VOCs) produced by leaf samples, (**A**) calla lily and (**B**) cabbage samples (double-distilled water (DDW)-black, Pc16- red, WPP14-green), following infiltration with bacteria, and incubation in airtight jars at 28 °C for 24 h, and adsorption onto a SPME fiber. The adsorbed compounds were analyzed using Agilent GC 7890A and 5975C MSD, and mass spectra were acquired in positive electron-impact (EI) scan mode (m/z 20–350). Identification of the VOCs was performed by comparing their mass spectra and retention times with the NIST/EPA/NIH Mass Spectral Database (NIST 05, National Institute of Standards and Technology, Gaithersburg, MD, USA; see Table 2 and Table 3). Representative chromatograms from two independent experiments are presented.

GC-MS was employed in an effort to identify VOCs in all six samples. In calla lily samples, a total of 16 VOCs were detected, 11 of which were identified (Figure 3 and Table 2). Four VOCs were common to all calla lily samples, while two, 4-methylheptane and 2, 4-dimethyl-1-heptene, were present only in calla lily + WPP14 and the calla lily control. Toluene was also identified, but only in the mock-inoculated calla lily control. Three VOCs, 4-methyloctane and two branched alkanes, were only detected following infection of calla lily with WPP14 samples. The headspace of calla lily + PC16 samples contained six VOCs that were unique to that interaction: methyl acetate, ethyl acetate, isopentyl alcohol, isobutyl acetate, isopentyl acetate and a compound similar to sandaracopimaradiene (Table 2).

In the cabbage samples, a total of 21 VOCs were detected, 12 of which were identified (Figure 3 and Table 3). Eleven of the VOCs detected were found in all of the cabbage samples, and three VOCs were detected for both cabbage + WPP14 and the cabbage control: two branched alkanes and 2, 6, 10, 14-tetramethyl heptadecane. The remaining seven VOCs were detected only following the inoculation of cabbage with WPP14: isopentyl alcohol, methyl disulfide and five unidentified compounds (Table 3).

Using 6 commercially available compounds that were suggested by the GC-MS library search we were able to confirm the identification of four compounds, namely methyl acetate, ethyl acetate, isopentyl alcohol and isobutyl acetate, which eluted at RT 2.36, 2.78, 3.80 and 4.28, respectively. These phytochemicals were only produced by calla lily that was challenged with PC16. Similarly, we confirmed four compounds at RT 2.10, 2.78, 3.84 and 4.20 as ethanol, ethyl acetate, isopentyl alcohol and 4-methyl heptane in all cabbage samples, respectively. Among these 4 compounds, isopentyl alcohol was specific to cabbage + WPP14 samples. Based on the FID signals, a marked increase in the amounts of ethanol and 4-methyl heptane was observed in cabbage leaves that were inoculated with WPP14.

### 3.4. Expression of Defense-related Genes in Calla Lily and Cabbage in Response to Pectobacterium Infection

**Figure 4:**
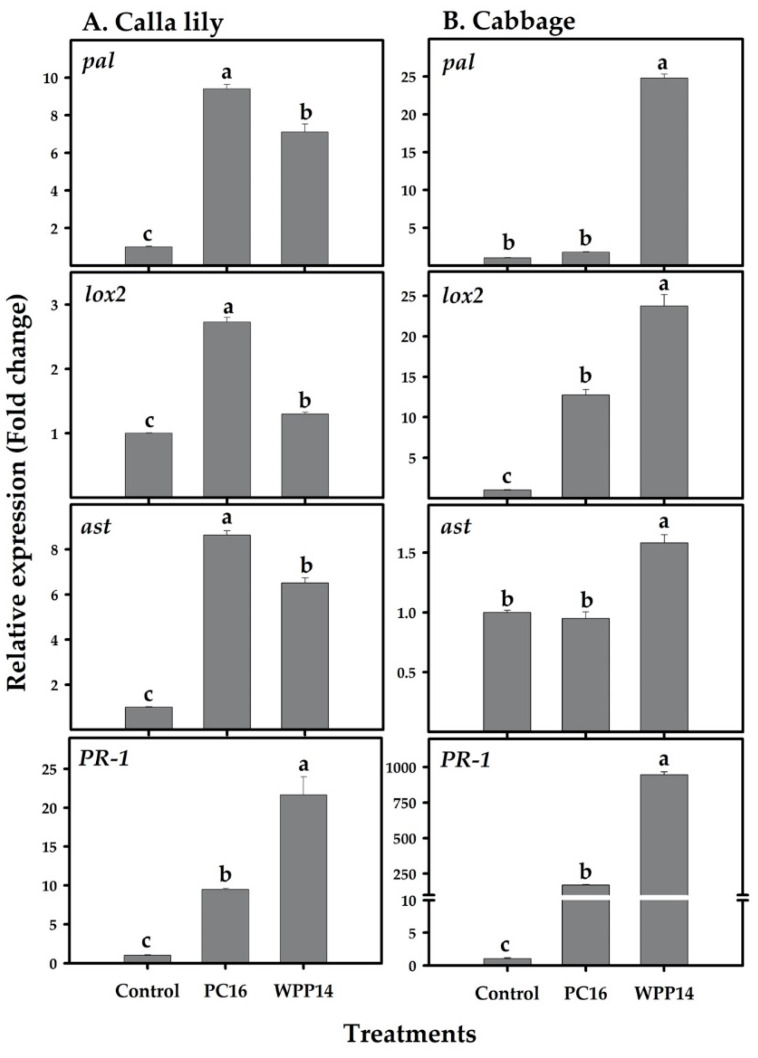
Expression of defense-related genes *pal*, *lox2*, *ast* and *PR-1* in (**A**) calla lily and (**B**) cabbage following infection with *Pectobacterium* strains PC16 or WPP14. Calla lily and cabbage leaves were infiltrated with overnight-grown *Pectobacterium* strains (100 µL, 10^7^ cfu/mL in sterile DDW) or sterile DDW. Twenty-four hours after infiltration, the transcript levels of these genes were determined by qRT-PCR using cDNA-specific primers and expressed relative to control. Experiments were repeated three times with three replicates per experiment. Bars represent mean ± SE of relative expression of each gene. Bars not labeled with the same letter (a–c) are significantly different from each other (one-way ANOVA with post hoc Tukey HSD test, *p* < 0.05).

Plant immune systems may be activated in response to biotic and abiotic stresses, including a pathogen attack. To better understand the pathways underlying the observed specificity of *Pectobacterium* strains to their hosts, we tested the expression of marker genes that are related to plant-defense pathways in two hosts: calla lily and cabbage. The expression of the genes for lipoxygenase (*lox2*), phenylalanine ammonia lyase (*pal*), aspartate aminotransferase (*ast*) and a pathogenesis-related gene (*PR-1*) were studied in response to infection with either PC16 or WPP14. In calla lily, all of these genes were significantly upregulated following infection with PC16 or WPP14. However, expression levels were higher following infection with PC16, as compared to WPP14, except for the gene *PR-1* (Figure 4A). In cabbage, the genes *lox2* and *PR-1* were significantly upregulated by both PC16 and WPP14, with greater expression in response to WPP14 infection. The genes *pal* and *ast* were only upregulated in response to infection with WPP14 (Figure 4B).

### 3.5. Effect of Host Phenolics on the Growth of the Pectobacterium Strains

**Figure 5:**
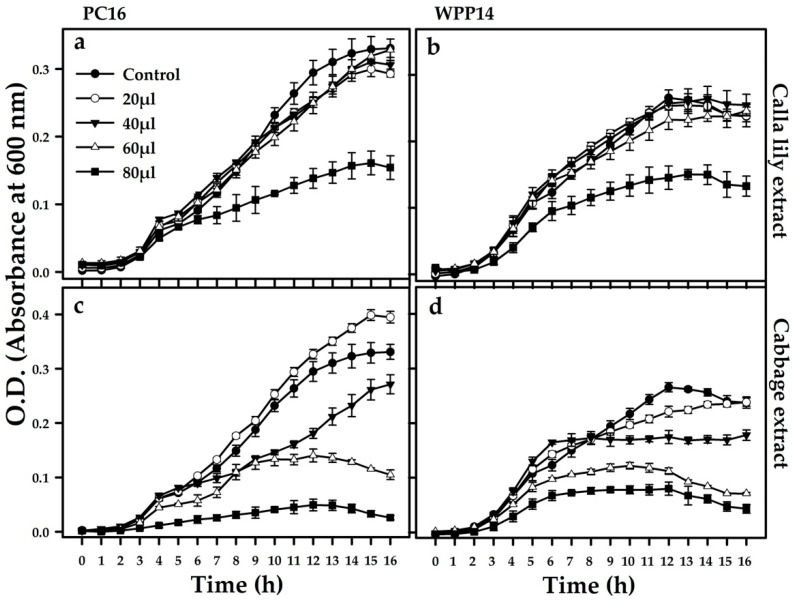
Effect of phenolic extracts from calla lily or cabbage leaves on the growth of *Pectobacterium* strains PC16 (**a**,**c**) and WPP14 (**b**,**d**). Bacteria were grown at 28 °>C for 16 h, with or without plant extract, under continuous shaking (150 rpm). Growth was assessed every hour and is presented as absorbance at 600 nm (OD_600 nm_). Experiments were repeated twice with four replicates per treatment; similar results were observed in the two repetitions (bar = mean ± SE; *n* = 8).

To study the potential effect of host extracts on virulence-related responses of the bacteria, plant extracts were added to the growth media. PC16 and WPP14 were then grown in the presence of increasing levels of extracts from each of the two hosts (Figure S3). Increasing the levels of calla lily extract (20, 40 or 60 µL added to 200 µL LB) did not affect bacterial growth (as shown by similar absorbance at 600 nm), however addition of 80 µL inhibited the growth of both bacterial strains (Figure 5a,b). Under all non-lethal conditions, PC16 reached a higher bacterial growth than WPP14. Both strains exhibited a dose dependent response to the cabbage extract (Figure 5c,d). At lower volumes, PC16 growth was enhanced by the cabbage extract. However, at the higher concentration of cabbage extract, the growth of PC16, and to a lesser extent WPP14, was significantly inhibited. The phenolic extracts of both hosts displayed a low level of antimicrobial activity, which was similar against both bacterial strains. To better mimic the plant environment, the growth of the two strains was also evaluated in MM and in the presence of non-lethal levels of the two extracts. Slower growth was observed for both bacteria in MM (Figure S4) and, in agreement with the previous results, the two strains grew similarly in MM in the presence of non-lethal levels of the two extracts (Figure S4).

### 3.6. Effect of Host Phenolic Extracts on Bacterial Exoenzyme Activity

**Figure 6:**
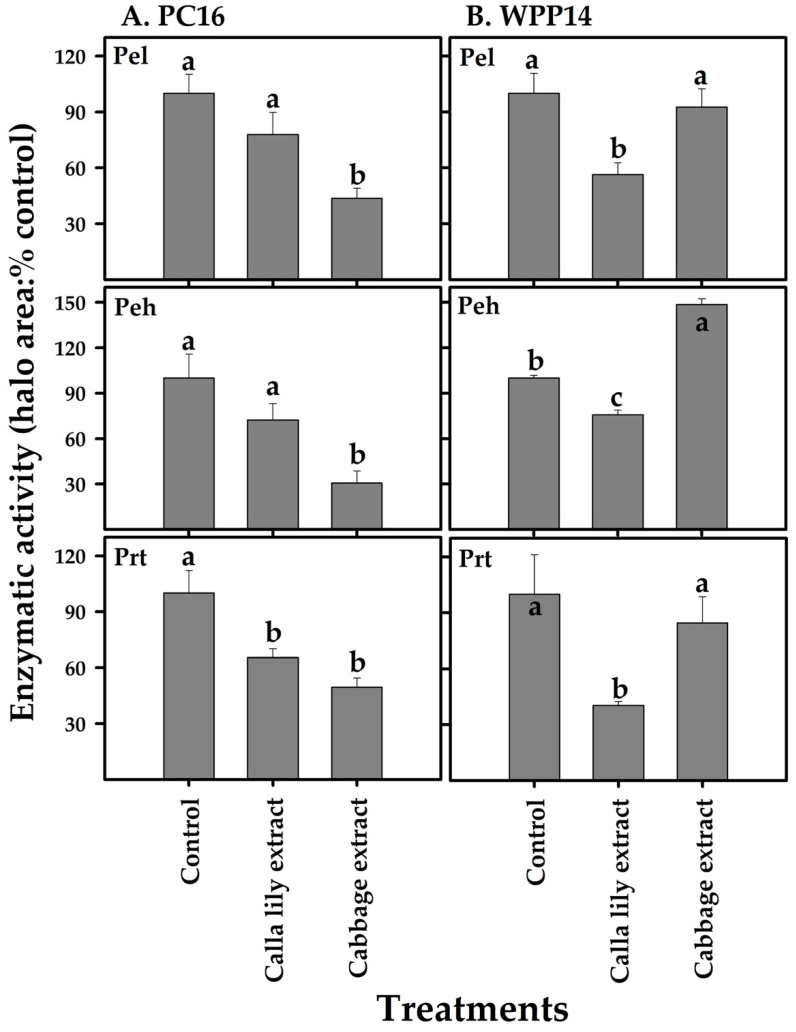
Effect of plant extracts (calla lily or cabbage) on the exoenzyme activity of *Pectobacterium* strains PC16 (**A**) and WPP14 (**B**). Enzymatic activity was measured 8 h after application of a non-lethal dose of plant extract to the microbial suspensions. Pel, Peh and Prt activities were determined based on the area of the haloes formed by substrate degradation. Results are expressed as the level of activity relative to that of the control (%). The experiments were repeated twice with eight replicates per treatment. Tukey–Kramer HSD test was used to test differences, bars labeled with different letter (a–c) are significantly different from each other (*p* < 0.05; bar = mean ± SE; *n* = 16).

The most important virulence factor of *Pectobacterium* spp. is its capability to secrete PCWDEs and macerate host tissues. The effect of non-lethal volume of host plant extracts on PCWDEs activity was assayed. While calla lily extract significantly reduced the activity of Pel, Peh and Prt in WPP14, the only effect it had on PC16 (Figure 6) was a significant reduction in the activity of Prt. The cabbage extract reduced the activity of all of the tested exoenzymes in PC16, but not in WPP14, in which Peh activity was significantly increased relative to the control. However, Pel and Prt activities were similar to control treatment (Figure 6).

### 3.7. Effect of Plant. Extracts on Biofilm Formation

**Figure 7:**
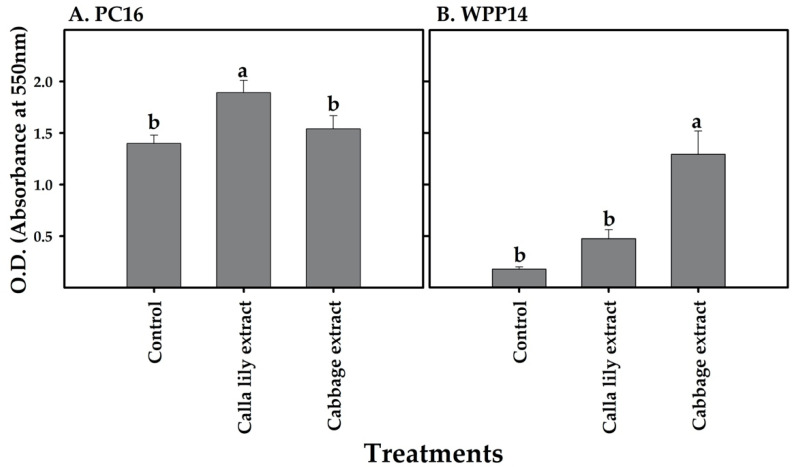
Effect of phenolic extracts from calla lily or cabbage leaves on the biofilm formation ability of *Pectobacterium* strains (**A**) PC16 and (**B**) WPP14. Biofilms were measured following the exposure of *Pectobacterium* strains to a non-lethal volume (40 µL extract/200 µL LB) of each host extract (or extraction buffer as control) for 72 h at 28 °C in glass test tubes. Biofilms were quantified by measuring absorbance at 550 nm (crystal violet bound to biofilm cells). Experiments were repeated twice with six replicates for each experiment. Tukey–Kramer HSD test was used to test the differences, bars not labeled with the same letter (a-b) are significantly different from each other (*p* < 0.05; bar = mean ± SE; *n* = 12).

Differential biofilm formation was observed for PC16 and WPP14 in response to a non-lethal volume (40 µL extract/200 µL LB) of the two host extracts. PC16 formed more biofilm in the presence of calla lily extract; however, cabbage extract did not affect its biofilm formation capacity (Figure 7A). In contrast, WPP14 had increased biofilm formation when exposed to cabbage extract (Figure 7B).

### 3.8. Expression of Pectobacterium Virulence Genes in Response to Host Phenolic Extracts

**Figure 8:**
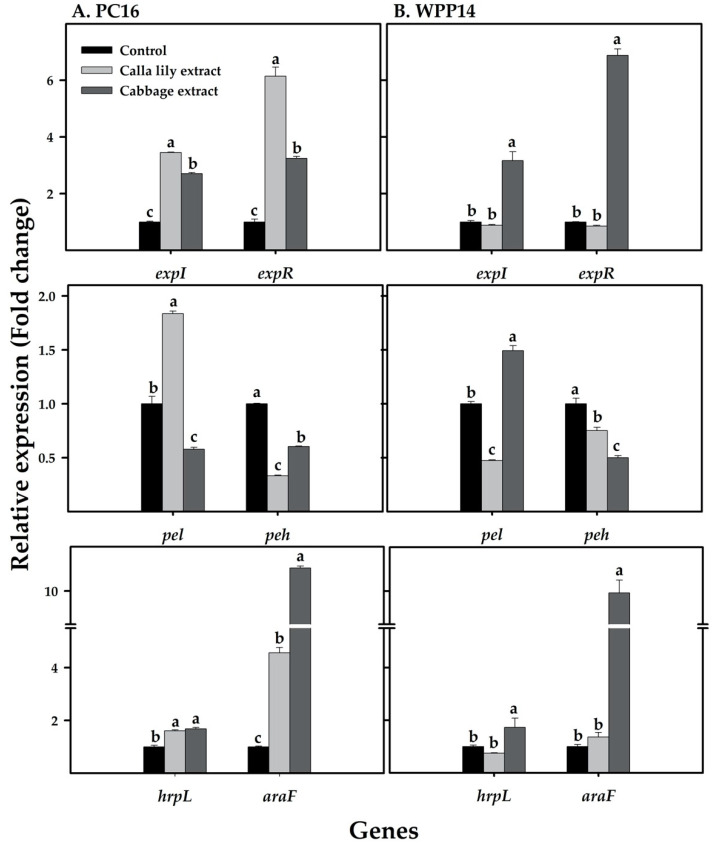
Expression levels of virulence-related genes in *Pectobacterium* strains (**A**) PC16 and (**B**) WPP14 in response to phenolic extracts from calla lily or cabbage leaves. The transcript levels of the examined genes were analyzed in DNA-free RNA from the bacterial strains PC16 and WPP14 after 8 h in LB medium with or without host extracts (control), at 28 °C under continuous shaking (150 rpm). The expression levels of *expI*, *expR*, *pel*, *peh*, *hrpL* and *araF* were quantified relative to control using qRT-PCR. Experiments were repeated three times with three replicates showing similar trends. Bars represents mean ± SE of relative expression of each gene. Tukey–Kramer HSD test was used to test the differences, bars not labeled with the same letter (a–c) are significantly different from each other (*p* < 0.05).

The expression of major virulence genes was assayed in the two bacterial strains in response to the studied plant phenolic extracts. The QS genes *expI* and *expR* were upregulated in PC16 in the presence of both host extracts, with higher fold change in response to calla lily extract. WPP14, showed higher expression levels of the QS genes only in response to the cabbage extract (Figure 8). The expression of *pel* in PC16 was upregulated in response to calla lily extract, whereas the cabbage extract significantly reduced its expression. An opposite response was observed in WPP14, in which *pel* expression was upregulated in the presence of cabbage extract and downregulated in the presence of calla lily extract. Unlike most of the responses described so far, the expression of *peh* was downregulated in both strains and no host specific effects on *araF* and *hrpL* was observed. PC16 showed an increase in the expression of both *araF* and *hrpL* in response to both plant’s extracts, while WPP14 presented higher expression of these genes only in response to the cabbage extract (Figure 8). The expression of the genes *expI*, *expR*, *pel* and *peh* was also evaluated in MM in the presence of both host extracts, to better mimic the plant ecosystem. Similar expression patterns were observed for these genes when the bacteria were grown in MM, however with lower fold change (Figure S5).

## 4. Discussion

*Pectobacterium* colonization of host plants cause severe damage to farmers, suppliers and consumers. This multifaceted process involves many factors, including pathogen virulence, interspecific interactions, host defense responses and environmental conditions. Little is understood about the level of specificity between certain pectobacteria with their plant hosts, or on the influence of such specificity on the outcome of these mutual associations. Here, several aspects of such host–pathogen interactions were studied. Host specificity has been suggested for *Pectobacterium* isolates from different hosts, but this hypothesis has not yet garnered conclusive support [13,17,31]. Host range studies based on genomic analyses, geographical distribution and hosts’ taxonomic classifications have revealed an ever-increasing phylogenetic and evolutionary associations within the genus [1,4,11,23,32,33,34]. Fewer studies have included virulence assays on different hosts, and mutual characterization of the differential interactions between certain pectobacteria and their natural hosts.

The most prominent virulence factor of soft rot bacteria is the secretion of PCWDEs, which are under the strict control of the QS system [35]. These enzymes are responsible for the development of disease symptoms such as wet lesions and the maceration of the plant tissue and, finally, the decay of plant organs. Accordingly, bacterial virulence traits were monitored in response to inoculation with phenolic extracts from the host leaves, which are known to contain most of the plant antimicrobial arsenal and which were previously shown to play a role in the defense response to *Pectobacterium* infection [25].

Differential infection patterns of the tested *Pectobacterium* strains were observed in infiltration assays and in a leaf disc assay. In both cases, each of the strains was more virulent on the host belonging to the plant clade from which it was isolated. The specificity to the host was observed as early as 3 h after inoculation using SEM. A short time that reflects the colonization process and the interface between the host and the pathogens [36]. The washing and dehydration process during the preparation for the SEM revealed what was apparently the initial attachment of the bacteria to the leaf surface and the formation of early structures such as fibers surrounding the cells. Such submicrometer-scale cell surface polymers are essential during cell adhesion and are typical to early establishment of biofilms [36]. Differential adhesion patterns with varying levels of cell aggregates were observed and found to be in line with the quantitative symptom assessment observed at 24 h after inoculation and bacterial CFU counting 3 h post inoculation.

Plants respond to biotic stress by activating defense mechanisms, including the production of complex blends of VOCs [29,30]. Specific VOCs have been shown to be part of a mechanism by which individual plants signal and communicate with neighboring plants. Some volatile phytochemicals are rather general and allow neighboring organs and other plants to recognize pathogens, attack them and elicit defense responses [29]. Here, specific VOCs were emitted, and characterized in the context of the interactions of PC16 and WPP14 with the two hosts. The results provided additional support for the specificity of the interaction, and indeed every plant with every pathogen produced a different array of VOCs. Calla lily leaves presented higher levels of VOC and a more complex VOC pattern in response to infiltration with PC16; whereas WPP14 triggered a high and complex response in cabbage leaves. Neither the opposite combinations nor the mock control led to the same plant response. Some of the volatiles produced by each compatible interface and analyzed by GC-MS and a library search, were recognized to be associated with typical defense responses, i.e., calla lily–PC16 interaction and the cabbage–WPP14 interaction. Some of the identified compounds, namely methyl- and ethyl- acetate and isopentyl alcohol, which were produced by calla lily in response to PC16, were previously suggested to be produced by the oxidation of monoterpenes by pathogens in a compatible plant–pathogen interaction [37]. Terpenes have been reported to play a key role in protecting plants from bacterial pathogens as in the case of rice against bacterial rice blight [38]. Isopentyl alcohol, dimethyl disulfide and several unidentified compounds were shown to be representative of cabbage–WPP14 unique interaction. Ethanol, ethyl acetate, isopentyl alcohol, dimethyl disulfide and octane were previously reported to be involved in the interaction of *Pectobacterium* spp. with different hosts [39,40]. To the best of our knowledge, this is the first report to propose that different *Pectobacterium* strains differentially modulate the VOCs production and emission by their hosts during compatible and non-compatible interactions.

Systemic acquired resistance (SAR) and induced systemic resistance (ISR) are the most recognized defense pathways in plants and are associated with biotrophic and necrotrophic pathogens, respectively [41]. Defense mechanisms activated in response to *Pectobacterium* infections are complex, as the pathogen deploys stealthy and brute-force modes of action depending on the prevailing environmental conditions [42,43,44]. Here, we assessed the expression of *lox2*, a typical ISR marker [45], that is also involved in the production of volatiles in relation to pathogen attacks [46]; the SAR marker *PR-1* [47]; *pal*, an enzyme involved in biosynthesis of salicylic acid and other phenolics [48]; and *ast*, which modifies the primary amino acid metabolism as part of defense responses in plants [49]. Significantly higher expression of *pal* and *lox2* was observed in calla lily when it was challenged with both strains. It is suggested that specificity may be expressed as the intensity of the gene expression of calla lily in response to PC16 or cabbage’s expression of *pal* and *lox2* upon WPP14 infection.

Synthesis of phenolics by these enzymes was previously demonstrated as part of ISR activation in calla lily and potato against pectobacteria [25,50]. These enzymes were also shown to be involved in the increased production of VOCs [46], and the expression of the relevant genes was shown to increase following infection of tomato and *A. thaliana* with *P. carotovorum* [51,52]. *PR-1* expression increased in both hosts upon infection with both strains, similar to the infections caused by necrotrophic pectobacteria or the necrotrophic fungus *Alternaria solani* in *A. thaliana* and tomato, respectively [51,53]. Since *PR-1* is related to SAR and programmed cell death, this response may in some cases enhance the infection. Another defense related gene, *ast,* was also shown to affect infection (lesion development) in overexpressing lines of *Arabidopsis* upon infection with *Botrytis cinerea* [49]. Here, upon infection of the host with its compatible strain a higher expression of *ast* was correlated with stronger development of disease symptom in both hosts, suggesting that the higher levels of expression of this gene leads to elevated disease.

Secondary metabolites including phenolics are part of an array of multifaceted defense mechanisms that plants activate in response to microbial pathogens and to insect herbivores [41,54]. Levels of either total or specific phenolics have been shown to increase following defense elicitation and/or necrotrophic pathogen infections, and are considered a part of plants’ defenses against *Pectobacterium* [25,55,56]. Here, the effects of phenolics containing extracts were tested on virulence factors and expression of virulence genes in both *Pectobacterium* strains. Exoenzyme activity (Pel, Peh and Prt) in both strains was assessed in the presence of non-inhibitory concentration of host phenolic extracts. Again, the unique nature of the interaction of each host with the different pathogen was clearly presented. The extracts inhibited exoenzyme activity in both strains in a reciprocal manner, with calla lily extract inhibiting the activity of Pel, Peh and Prt in WPP14 and cabbage extract reducing the activity of the same enzymes in PC16.

Biofilm formation another important trait for successful colonization of hosts by plant-pathogenic bacteria [57,58], also increased in a differential manner with each strain responding to the extract from its favored host. Increased biofilm formation was seen in *Pseudomonas aeruginosa* and *Agrobacterium tumefaciens* in the presence of non-lethal concentrations of phenolic compounds [59]. Differential biofilm formation of the two *Pectobacterium* strains may be another factor affecting host specificity.

Finally, the effects of the host extracts on the expression of bacterial virulence genes was evaluated. The genes *expI* and *expR* major components of the AI-1 quorum-sensing (QS) circuit in *Pectobacterium* [60], largely control virulence by regulating PCWDEs, biofilm formation and T3SS [43,61]. Differential expression of these genes was observed for both strains in response to the application of the extracts from their respective host. A similar pattern was observed for *pel* gene in both strains. Similar results were observed in *Pseudomonas syringae* pv. *phaseolicola* NPS3121 and *Xanthomonas campestris* pv. *campestris* following exposure to extracts from their own hosts common bean (*Phaseolus vulgaris*) and cabbage, respectively [62,63]. The expression of *pel* was quite congruent with the differences in enzymatic activities of both strains. The varying expression of *Peh* suggests that this enzyme is also regulated by other factors, as was suggested for the low-cell-density environment that exists mainly at earlier stages of infection [64]. Other genes like *hrpL* and *araF* were significantly upregulated by cabbage phenolics in both strains. The calla lily extract affected the expression levels of these genes only in PC16. HrpL protein, a product of the *hrpL* gene, is an alternative sigma factor that regulates the expression of structural components of T3SS and other genes that have been associated with *Erwinia amylovora* biofilm formation [57]. Finally, although PCWDEs, biofilm formation and the expression of virulence genes in *Pectobacterium* spp. were shown not to depend on the growth medium, we also examined some of these traits in MM, since it was suggested to be a better mimic of the host environment [65]. The differential effect of the plant host extracts was conserved for both strains in MM. Overall, the results support host specialization by certain pectobacteria.

## 5. Conclusions

Pectobacteria are commonly recognized as broad-host-range pathogens. Here, high levels of recognition, primarily based on the production of specific molecules, were demonstrated in the interactions of two *Pectobacterium* species with their typical hosts. Not only could each bacterial strain sense its “favored” host and express different virulence factors in response, the plant hosts have also identified the colonizing strain and activated different defense mechanisms. Wider transcriptomic work is required to better characterize the specific genes and signals that may be involved in both host and pathogen specialization.

## Figures and Tables

**Table 1:** Primers used for qRT-PCR.

Genes.	Forward Primer (5′→3′)	Reverse Primer (5′→3′)
A. PC16 and WPP14		
*recA* (reference gene)	CAG CAT CGA TGA ACG CAC AG	GGT TTA CCG ATG GGC CGT AT
*pel*	CTT CTT CAT GGC CGA TCC CA	CAA CGG ACT GTG GCT GAT TG
*peh*	TAC CGC TAC GAG TAC GAC GA	GAT CCC ACC AGC TCA CCT TT
*expI*	TAC AAT AGC GGC AGG CAC TC	TGA GAA TCA GGA AGC ATT GGC
*expR*	TGA GGT CAT GAG ATG TCG CC	TTA TGC CGT CGT AGC GAT CC
*hrpL*	AAC GTC GTC ATG GTT GCT GA	AAA CAC ATC GAA CCG ACC CC
*araF*	GGC TGA TTA TCG GTA TGA ACG	CCT GTT GCC TGA CCT TTG
B. Calla lily		
*actin* (reference gene)	GAC TCA AAT CAT GTT AGA GAC ATT CAA	GTA CGG CCA CTG GCA TAG A
*pal*	GAC CTC GTC CCG CTC TCC TAC A	CTC CAC AGC AGA GAC GTG GTG AC
*lox2*	CAT CAA GCT GCC AAG AGG TT	GCA ACC AAG AAA ATC CGT CT
*ast*	GCC AGT GGT GAT CCT GAG AG	GGA TGC TGA GGC AAC CTA CT
*PR-1*	GGT AGA ACC TCT TCT GGG GAT G	AGT TGC TTC GGT AGT CGT AGT AC
C. Cabbage		
*tubulin* (reference gene)	GGC TTC ACC ATT TAC CCT TC	TCC AAG AGG ACA GCA ACA TC
*pal*	ATC CCT AAC CGC CGA AGA G	TCC GCT AAC ACC GAT TGA AC
*lox2*	GCT CTC CGT TCC ATA AAC CA	TAT TTG ACT GTG ATG CCG TGA
*ast*	TCC AAC CAC CAC AAC ATC TG	CAC ATC ATC CAT CAA TCC TTT G
*PR-1*	GGA GAG AAC ATC GCT TGG AG	CAC ACA ACC TGC GTA TAG TGG

**Table 2:** VOCs collected from the headspace of calla lily samples inoculated with PC16 or WPP14 and the corresponding control (DDW), as identified by GC-MS.

RT (min)	Identified Volatile Compounds	Calla lily Control	Calla lily + PC16	Calla lily + WPP14
2.36	Methyl acetate		**+**	
2.78	Ethyl acetate		**+**	
3.80	Isopentyl alcohol		**+**	
4.20	4-Methylheptane	+ ^1^		**+**
4.25	Toluene	**+**		
4.28	Isobutyl acetate		**+**	
5.09	2,4-Dimethyl-1-heptene	**+**		**+**
5.38	4-Methyloctane			**+**
5.51	Isopentyl acetate		**+**	
7.13	Branched alkanes	**+**	**+**	**+**
7.18	Branched alkanes	**+**	**+**	**+**
7.70	Dibutoxy-methane	**+**	**+**	**+**
7.96	Branched alkanes			**+**
8.01	Branched alkanes			**+**
10.09	2,6,10,14-tetramethyl heptadecane	**+**	**+**	**+**
16.1	Similar to sandaracopimaradiene		+	

^1^ “**+**” indicates presence of that compound in the given sample.

**Table 3:** VOCs collected from the headspace of cabbage samples inoculated with PC16 or WPP14 and the corresponding control (DDW), as identified by GC-MS.

RT (min)	Identified Volatile Compounds	Cabbage Control	Cabbage + PC16	Cabbage + WPP14
2.10	Ethanol	+ ^1^	**+**	**+**
2.78	Ethyl acetate	**+**	**+**	**+**
3.65	3-Hydroxy-2-butanone	**+**	**+**	**+**
3.84	Isopentyl alcohol			**+**
4.0	Methyl disulfide			**+**
4.20	4-Methylheptane	**+**	**+**	**+**
4.58	Octane	**+**	**+**	**+**
4.88	2,4-dimethylheptane	**+**	**+**	**+**
5.10	2,4-dimethylheptene	**+**	**+**	**+**
5.36	4-methyloctane	**+**	**+**	**+**
7.13	Branched alkanes	**+**	**+**	**+**
7.18	Branched alkanes	**+**	**+**	**+**
7.71	Dibutoxy-methane	**+**	**+**	**+**
7.96	Branched alkanes	**+**		**+**
8.01	Branched alkanes	**+**		**+**
8.26	Not identified			**+**
8.34	Not identified			**+**
9.73	Not identified			**+**
10.09	2,6,10,14-tetramethyl heptadecane	**+**		**+**
10.37	Not identified			**+**
11.20	Not identified			+

^1^ Sign “**+**” indicates presence of that compound in the given sample.

## Supplementary Materials

Supplementary materials can be found at https://www.mdpi.com/2076-2607/8/10/1479/s1, Figure S1: Leaf discs of calla lily or cabbage leaves inoculated with *Pectobacterium* strains, Figure S2: Assessment of bacterial colonization on leaves, 3 h post inoculation of *P. carotovorum* WPP14 or *P. aroidearum* PC16 onto calla lily or cabbage leaf surface, Figure S3: HPLC chromatograms of phenolics extracted from Calla lily and Cabbage, that served for the assays, Figure S4: Growth of *Pectobacterium* strains PC16 and WPP14 in MM in the presence of non-lethal volumes (40 µL extract/200 µL LB) of phenolic extracts from calla lily or cabbage leaves, Figure S5: Expression levels of virulence genes (*expI*, *expR*, *pel* and *peh*) in *Pectobacterium* strains PC16 and WPP14 in MM in the presence of phenolic extracts from calla lily and cabbage leaves.
