# Supplementary material for: Host Specificity and Differential Pathogenicity of Pectobacterium Strains from Dicot and Monocot Hosts
Source: Microorganisms. 2020 Sep 26;8(10):1479. doi: 10.3390/microorganisms8101479 (PMC7599833; doi:10.3390/microorganisms8101479)
Supplement: Supplementary file 1 [file microorganisms-08-01479-s001.pdf]

microorganisms

Article

Host Specificity and Differential Pathogenicity of *Pectobacterium* Strains from Dicot and Monocot Hosts

Nirmal Khadka [1,2], Janak Raj Joshi [1,2], Noam Reznik [2], Nofar Chriker [1,2], Adi Nudel [1], Einat Zelinger [1], Zohar Kerem [1,*] and Iris Yedidia [2,*]

[1] Department of Biochemistry, Food Science and Nutrition, The Robert H. Smith Faculty of Agriculture, Food and Environment, The Hebrew University of Jerusalem, POB 12, 7610001, Rehovot, Israel; nirmal.khadka@mail.huji.ac.il (N.K.); janakra.joshi@mail.huji.ac.il (J.R.J.); nofar.tsriker@mail.huji.ac.il (N.C.); nudel.adi@gmail.com (A.N.); einat.zelinger@mail.huji.ac.il (E.Z.)

[2] Institute of Plant Sciences, Agricultural Research Organization, Volcani Center, POB 15159, 752880, Rishon Lezion, Israel; noamr@volcani.agri.gov.il

* Correspondence: zohar.kerem@mail.huji.ac.il (Z.K.); irisy@volcani.agri.gov.il (I.Y.); Tel.: +972-3-968-3387 (I.Y.)

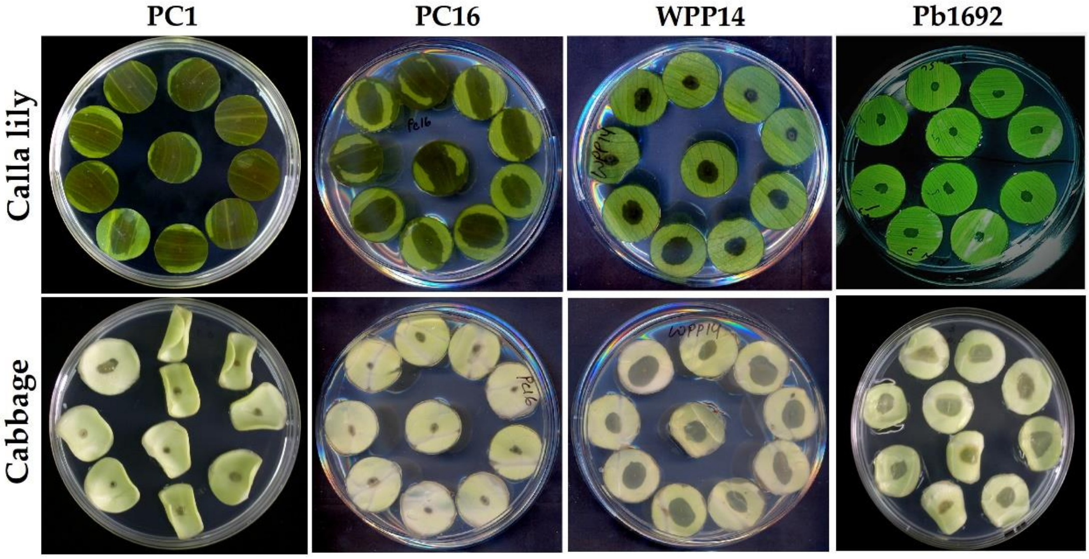

Figure S1. Virulence assessment on calla lily or cabbage leaf discs in response to inoculation with *Pectobacterium* strains isolated from monocot or dicot hosts. Leaf discs of calla lily or cabbage were pierced in the center and inoculated with *Pectobacterium* strains *P. aroidearum* PC1 and PC16, *P. carotovorum* WPP14 and *P. brasiliense* Pb1692 (10 µL of 10^8 cfu/mL). Infection was measured as percentage of decayed tissue at 15 h after inoculation. Experiments were repeated twice with 20 replicates for each strain/host combination, with similar results observed across the replicates.

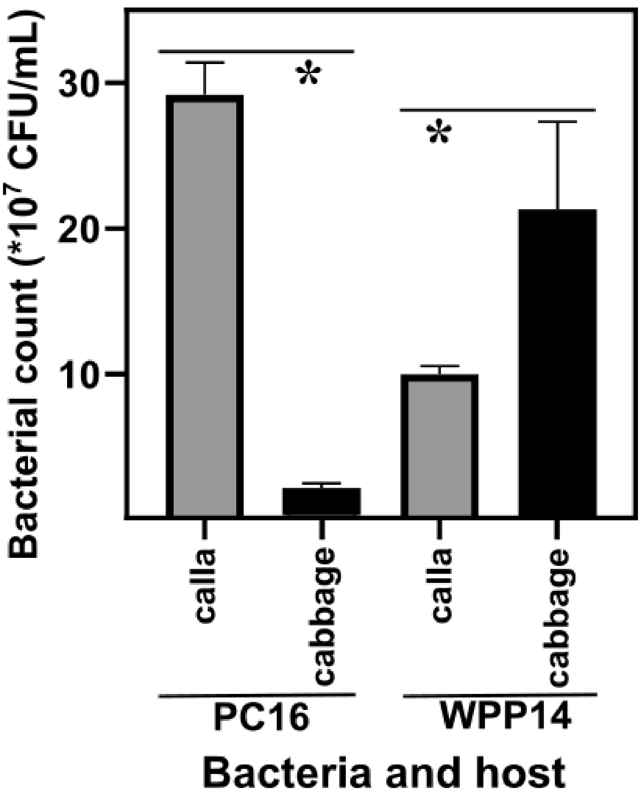

Figure S2. Assessment of bacterial colonization on calla lily and cabbage leaf surfaces, 3 h post inoculation of *P. carotovorum* WPP14 or *P. aroidearum* PC16, expressed as CFU/mL. Disinfected calla lily and cabbage leaves were inoculated with 10 μL (10^8 cfu/mL, placed on leaf surface without injuring tissue) of overnight grown bacterial cultures and incubated at 28°C for 3 h, inside an airtight box. Leaf samples (4 mm × 4 mm), from bacterial inoculation site, were gently rinsed with DDW, and sections (4 mm × 4 mm) from 5 leaves each with two replicates were excised and grinded in 200 μL sterile DDW. Serial dilutions were made and plated onto LB agar for bacterial assessment. Bars represents mean ± standard errors (SE) of bacterial CFU/mL. Treatments labeled with * are significantly different ($p < 0.05$). Student's t-test was used to analyze significance by GraphPad Prism 8.0.

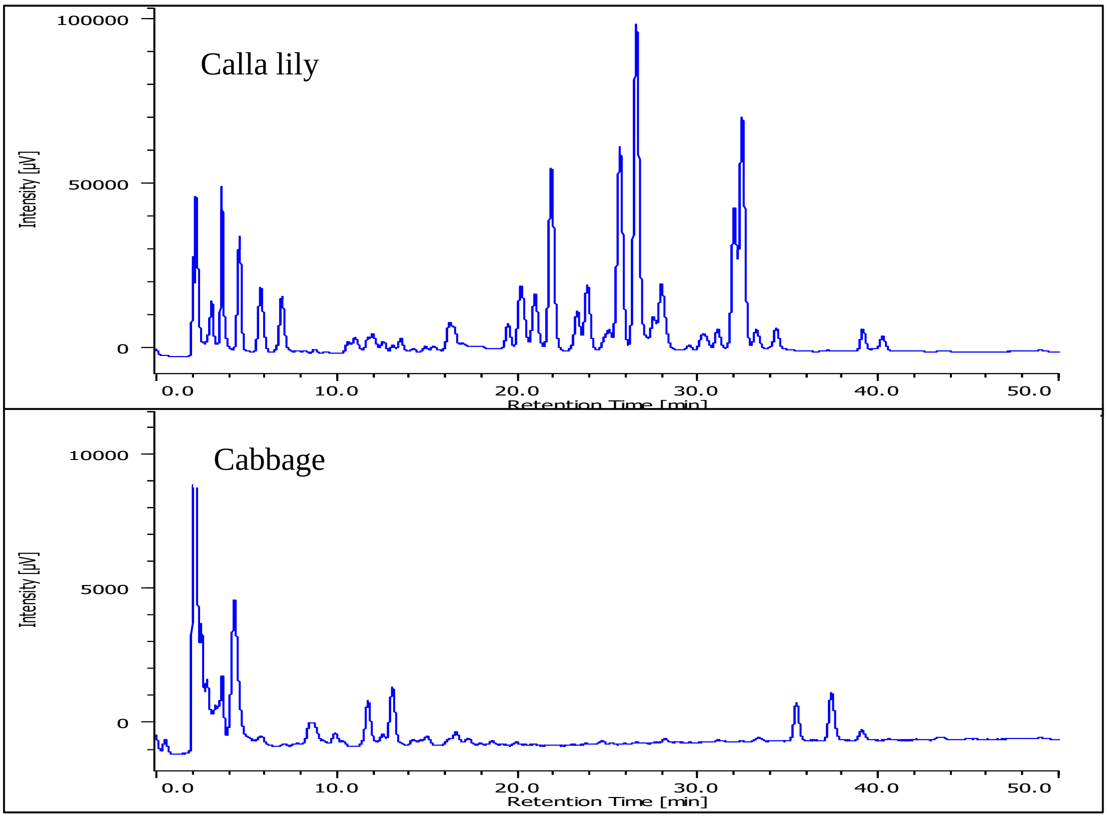

Figure S3. HPLC chromatograms of phenolics extracted from Calla lily (top) and Cabbage (bottom), that served for "bacterial responses to plant extracts" assays. The HPLC (Thermo Separation Products, San Jose, CA, USA) consisted of an AS3000 autosampler, a 100 µL injector, a P3000 pump, a UV6000 diode array detector, and a 25 × 4.6 mm reverse-phase Luna2 C18 column (Phenomenex, USA). A linear gradient using water and methanol at a flow rate of 1 mL/min was used, following 2 min at 40% methanol, and reaching 55% methanol in 58 min, phenolics were monitored at 270 nm.

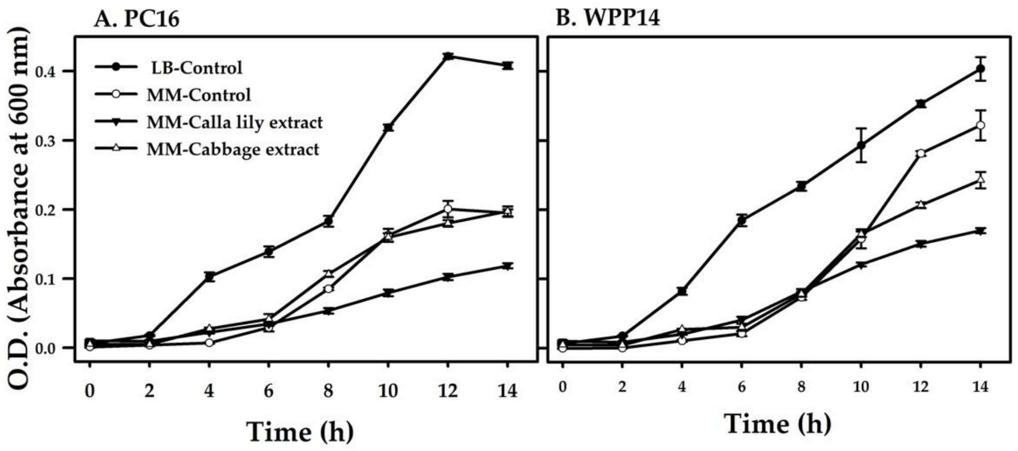

Figure S4. Growth of *Pectobacterium* strains **(A)** PC16 and **(B)** WPP14 in MM in the presence of non-lethal volume (40 μL extract/200 μL LB) of phenolic extracts from calla lily or cabbage leaves. Bacteria were grown at 28°C for 14 h, with or without host extract, under continuous shaking (150 rpm). Growth was assessed every 2 h and is presented as absorbance at 600 nm (OD 600). Experiments were repeated twice with 4 replicates for each treatment, with similar results observed in each repetition (bar =mean ± SE; *n* = 8).

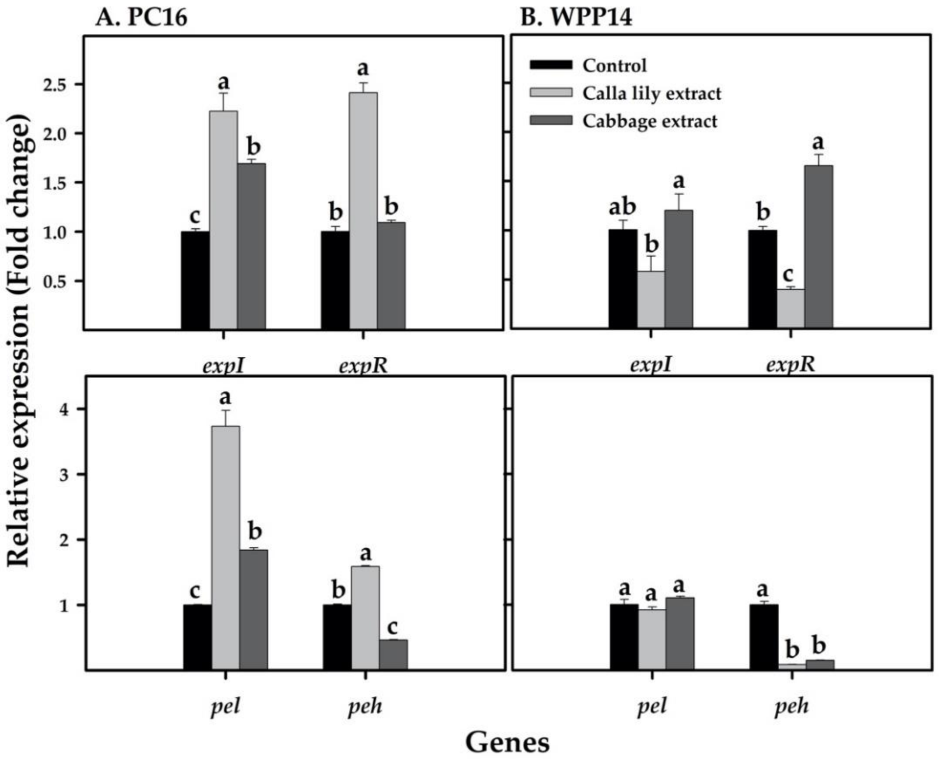

Figure S5. Expression levels of virulence genes (*expI*, *expR*, *pel* and *peh*) in *Pectobacterium* strains **(A)** PC16 and **(B)** WPP14 in the presence of phenolic extracts from calla lily and cabbage leaves. The transcripts levels of the tested genes were analyzed in DNA-free RNA from the bacterial strains PC16 and WPP14 after 12 h in minimal medium (MM) with or without (as control) phenolic extracts, at 28 °C under continuous shaking at 150 rpm. The expression levels were quantified relative to control using qRT-PCR. Bars represents mean ± SE of relative expression of each gene. Tukey-Kramer HSD test was used to test the differences and bars not labeled with the same letter are significantly different from each other ($p \leq 0.05$).
